# YAP increases response to Trastuzumab in HER2-positive Breast Cancer by enhancing P73-induced apoptosis

**DOI:** 10.7150/jca.48535

**Published:** 2020-09-25

**Authors:** Lanqing Cao, Min Yao, Hironobu Sasano, Ping-Li Sun, Hongwen Gao

**Affiliations:** 1 Department of Pathology, The Second Hospital of Jilin University, Changchun, Jilin 130041, China.; 2 Department of Pathology, Tohoku University School of Medicine and Tohoku University Hospital, 2-1 Seiryo-machi, Aoba-Ku, Sendai, Miyagi 980-8575, Japan.

**Keywords:** Tumor progression, Chemotherapy, Neoadjuvant therapy, Breast cancer, Protein kinase B/AKT

## Abstract

The role of the Yes-associated protein (YAP) in oncogenesis and progression of breast cancer remains controversial. Meanwhile, development of therapeutic resistance to trastuzumab, a common breast cancer treatment administered after chemotherapy, is a significant challenge in the treatment of HER2-positive breast cancer. We, therefore, analyzed the role of YAP in trastuzumab resistance in HER2-positive-breast carcinoma cells *in vitro* and evaluated the status of YAP and related proteins in patient-derived breast carcinoma tissues by immunohistochemistry. YAP expression was observed in both BT474-TS (trastuzumab-sensitive) and BT474-TR (trastuzumab-resistant) cells. Treatment with trastuzumab increased expression of nuclear-YAP (N-YAP) in BT474-TS cells, whereas BT474-TR cells showed a decrease in N-YAP expression following trastuzumab treatment. YAP silencing significantly reduced trastuzumab-induced inhibitory effects in BT474-TS cells. YAP-silenced cells also showed decreased apoptosis and significantly lower p73 levels following trastuzumab treatment. Combined protein kinase B (AKT) inhibitor-trastuzumab treatment significantly inhibited BT474-TR cell proliferation, resulting in increased N-YAP and p73 expression, as well as apoptosis. In both paclitaxel, doxorubicin and cyclophosphamide (TAC)-treated, and docetaxel, carboplatin, and trastuzumab (TCbH)-treated groups; the pathological complete response (pCR) ratios were inversely correlated with p-AKT status in biopsy specimens, while YAP and p73 status were positively correlated with the pCR ratio in the biopsy specimens of the TCbH group. Our results show that YAP is involved in trastuzumab resistance in HER2-positive breast carcinoma cells and that YAP and AKT may be developed as prognostic markers of neoadjuvant trastuzumab therapy in patients with HER2-positive breast cancer.

## Introduction

Trastuzumab is an anti-HER2 monoclonal antibody used for targeted cancer therapy either alone, or in combination with other therapeutic drugs, in patients with HER2-positive breast or gastric carcinoma 1. Adjuvant chemotherapy with trastuzumab substantially enhances the efficacy of chemotherapy in HER2-positive breast cancer. However, although trastuzumab is one of the most effective targeted cancer therapies for patients with HER2-positive breast cancer, development of clinical resistance to trastuzumab has been observed in a significant number of patients undergoing adjuvant therapy 2-4, emphasizing the importance for understanding the underlying mechanisms associated with trastuzumab resistance in patients with breast cancer. Various potential anti-HER2 resistance mechanisms have been described as underlaying the activation of the HER2 pathway, or its downstream signaling, via pathway redundancy or stimulation of alternative survival pathways 5. Some of these mechanisms include incomplete blockade of the HER2 receptor, which subsequently activates the compensatory mechanisms within the HER family (such as HER3) and the alternative receptor tyrosine kinases or other membrane receptors outside the HER family (such as insulin-like growth factor 1 receptor and MET) 6, 7, as well as altering the downstream signaling pathways, such as hyperactivation of the PI3K/AKT/mTOR pathway 8, 9, by reducing the levels of tumor suppressor genes (*PTEN* and *INPP4B*) or inducing mutations in PIK3CA (phosphatidylinositol-4,5 bisphosphate 3-kinase catalytic subunit) 10.

Yes-associated protein (YAP), a downstream effector molecule of the Hippo signaling pathway, serves as a transcriptional coactivator and is reportedly expressed in many human malignancies 11-13, including breast cancer 14. Nuclear-YAP (N-YAP) interacts with transcription factors and promotes cancer cell proliferation, while maintaining stemness and metastasis 15-19. Furthermore, its nuclear abundance was reported to correlate with tumor progression and decreased survival in patients with breast cancer 20-22. In contrast, several studies have reported tumor suppressor roles for YAP in patients with breast cancer and have demonstrated a significant loss of YAP in breast cancer 23, 24. Taken together, these results support the presence of homeostatic machinery functioning to regulate the intracellular "tug-of-war" between the oncogenic and tumor suppressor factors that tightly regulate the expression of YAP. Moreover, dysregulation of YAP expression, or alteration of components associated with the multiple signaling pathways converging on these factors, serves as important mechanisms of resistance to chemotherapy and target therapy. Matrix-dependent resistance to lapatinib (HER2 inhibitor) has been linked to the expression of YAP and TAZ, which transduce substrate rigidity signals from the plasma membrane to the nucleus 25, 26. Conversely, a recent study proposed YAP as a mediator of chemotherapy sensitivity in pancreatic cancer 27. Hence, there appears to be a role for YAP in promoting cancer cell susceptibility to certain chemotherapeutic regimens; however, this modulatory capacity has proven heterogenous and is likely influenced by both context-specific and drug-specific mechanisms.

Additionally, we, along with others, have demonstrated that increased YAP status in carcinoma cells serves as a predictor for improved survival in patients with breast cancer 14, 28, 29. Hence, in the current study, we examined whether YAP silencing affects the efficacy of trastuzumab in HER2-positive trastuzumab-sensitive and -resistant breast carcinoma cell lines, as well as in pathology specimens from patients with breast cancer. We also aimed to investigate the possible mechanisms underlying the development of trastuzumab resistance to improve the therapeutic efficacy of trastuzumab in patients with HER2-positive breast cancer.

## Material and Methods

### Cell lines

Human breast carcinoma cell lines, BT474-TS (trastuzumab-sensitive) and BT474-TR (trastuzumab-resistant) (ER positive/HER2 positive), were obtained from the American Type Culture Collection (ATCC, Manassas, VI, USA) 30, 31. BT474-TR is derived from the BT474-TS cell line. Human breast carcinoma cell lines, MCF-7 (ER positive/HER2 negative) and MDA-MB-231 (ER negative/HER2 negative), were kindly provided by Prof. Jun Lu (The Institute of Genetics and Cytology, Northeast Normal University, Changchun, China). All cell lines were cultured in Dulbecco's modified eagle medium (DMEM) supplemented with 10% fetal bovine serum (FBS) at 37 °C in a humidified atmosphere incubator with 5% CO_2_.

### Western blot

Cells were lysed using radioimmunoprecipitation assay (RIPA) buffer (Beyotime LLC, Jiangsu, China), vortexed for 30 s, and centrifuged at 14,000 *× g* for 5 min at 4 °C. The supernatants were then collected and stored at -20 °C. Nuclear protein extraction was performed using the ProteinExt Mammalian Nuclear and Cytoplasmic Protein Extraction Kit (TransGen Biotechnology, Beijing, China) according to the manufacturer's instructions. Whole-cell lysates were resolved by sodium dodecyl sulfate polyacrylamide gel electrophoresis (SDS-PAGE), and the proteins were blotted onto a nitrocellulose membrane. The expression of various proteins was detected using the following primary antibodies: p73 (Cat. #5B429; NOVUS, St. Louis, MI, USA; 1:500 dilution in phosphate-buffered saline [PBS]), β-actin (Cat. #HC201-01; TransGen Biotechnology LLC; 1:1,000 dilution in PBS), YAP (Cat. #14074; Cell Signaling Technology, Danvers, MA, USA; 1:1,000 dilution in PBS), as well as AKT (Cat. #4691; 1:1,000 dilution in PBS), phosphorylated AKT (p-AKT, Ser473; Cat. #4060; 1:2,000 dilution in PBS), caspase-3 (Cat. #9662; 1:1,000 dilution in PBS), cleaved caspase-3 (Asp175; Cat. #9661; 1:1,000 dilution in PBS), PARP (Cat. #9532; 1:1,000 dilution in PBS), cleaved PARP (Asp214; Cat. #5625; 1:1,000 dilution in PBS) and GAPDH (Cat. #5174; 1:1,000 dilution in PBS) purchased from Cell Signaling Technology. Finally, the blots were visualized using an electrochemiluminescence system (TECAN, Beijing, China); western blot results were quantified using Image J software.

### Determination of cell viability

Cells were seeded in 96-well plates at a density of 5.0 × 10^3^ cells per well, cultured for 24 h, and treated with trastuzumab (Roche Ltd., Basel, Switzerland) at various concentrations for 48 h or the indicated durations. The cells were then incubated with 10 μL cell counting kit-8 (CCK-8) (Beyotime Biotechnology LLC, Shanghai, China) solution for 30‒45 min at 37 °C. Absorbance at 450 nm was detected using a microplate reader (Bio-Rad Model 680). The cell inhibitory index was calculated as [1-(A450 sample-A450 blank) / (A450 control-A450 blank)] × 100%.

### Determination of apoptosis

Control or treated cells were collected and stained with Annexin V-PE/7-AAD (Becton Dickinson, Franklin Lakes, NJ, USA). Apoptotic cell death was measured by counting the ratio of the AV-phycoerythrin positive cells, as determined by flow cytometry (Beyotime Biotechnology LLC).

### Quantitative real-time polymerase chain reaction (qRT-PCR)

Total RNA was isolated from 5 × 10^6^ cells using TRIzol reagent (Invitrogen, CA, USA) according to the manufacturer's instructions. Total RNA concentration and purity were analyzed in duplicate samples using a Nanodrop ND-2000 spectrophotometer (Thermo Fisher Scientific, MA, USA). Next, cDNA was synthesized from the qualified RNA using an RT-PCR reverse transcription kit (TransGen Biotechnology), and 1,000 ng of total RNA was reverse transcribed into cDNA under the following conditions: 25 °C for 10 min, 42 °C for 30 min, and 85 °C for 5 s, as per the manufacturer's recommendation. The cDNA was then stored at -20 °C until use. PCR was performed using a PCR kit (TransGen Biotechnology), and the PCR products were electrophoresed on 1.5% agarose gels. Quantitative PCR was carried out with either Taq-Man or SYBR Green PCR reagents on an ABI Prism 7300 detection system (all from Applied Biosystems, Foster City, CA, USA). The reaction program was as follows: 95 °C for 3 min, followed by 40 cycles of 95 °C for 30 s, 55 °C for 20 s, and 72 °C for 15 s. *GAPDH* served as an internal control, and the relative mRNA levels were calculated using the 2^-ΔΔCt^ method. The following primers were synthesized by Sangon Biotech (Shanghai, China): *YAP* (qRT-PCR)-forward: 5'-TAGCCCTGCGTAGCCAGTTA-3'; *YAP* (qRT-PCR)-reverse: 5'-TCATGCTTAGTCCACTGTCTGT-3'; *GAPDH* (qRT-PCR)-forward: 5'-GGAGCGAGATCCCTCCAAAAT-3'; *GAPDH* (qRT-PCR)-reverse: 5'-GGCTGTTGTCATACTTCTCATGG-3'.

### Transfection

The YAP-shRNA plasmid pLKO.1-shYAP1#1-Puro (#P1309; MiaoLingBio, Changchun, China), which expressed green fluorescent protein (GFP) (5'-TACAACAGCCACAACGTCTAT-3') was constructed. The YAP overexpression plasmid pCDNA3.1-YAP1-3×FLAG (#P8205; MiaoLingBio) and p73 overexpression plasmid HA-p73α-pCDNA3 (#P0552; MiaoLingBio) were transfected into BT474-TS and BT474-TR cells. A functional, non-targeting shRNA and an empty vector were used as a control. The target shRNA sequences for human YAP were as follows: shRNA-33: 5'-GGAATTGAGAACAATGACGAC-3'; shRNA-34: 5'-GGAGATGGAATGAACATAGAA-3'; shRNA-35: 5'-GCAGCAGAATATGATGAACTC-3'; shRNA-36: 5'-GGATACAGGTGATACTATCAA-3'.

### Immunohistochemistry

To study protein expression in breast cancer tissues, paraffin-embedded tissue sections were deparaffinized and incubated with antibodies against YAP (Cat. #14074; 1:400 dilution in PBS), AKT (1:300 dilution in PBS), and p-AKT (1:100 dilution in PBS) purchased from Cell Signaling Technology, as well as p73 (1:200 dilution in PBS) (NOVUS, St. Louis, MI, USA), followed by biotin-conjugated secondary antibody, using the PV-9001 IHC kit (Zhongshan Golden Bridge Biotechnology LLC, Beijing, China) at 37 °C for 30 min. The color reaction was performed using a 3,3'-diaminobenzidine kit (Zhongshan Golden Bridge Biotechnology LLC) according to the manufacturer's instructions.

### Review and scoring of immuno-stained tissue sections

The immuno-stained tissue sections were scored independently and reviewed by two pathologists to determine the percentages and intensity of immunostaining, as described previously 32. A final numerical score (FS = P × I) was calculated for each tissue sample by multiplying the intensity (I) score (0, negative; 1, weak; 2, moderate; 3, intense staining) by the percentage (P) of positively stained tumor cells (0-100). The FS ranged from 0 to 300. Next, the median FS was used as the cut-off value to score each case as positive or negative during statistical analysis.

### Patients and tissue specimens

This study was approved by the Ethics Committee of The Second Hospital of Jilin University (Jilin, China) (IRB approval number: 2019028). The study was exempted from the requirement of written informed consent due to its retrospective nature.

A total of 23 HER2-negative and 14 HER2-positive patients with breast cancer who underwent neoadjuvant chemotherapy at the Second Hospital of Jilin University between January 2016 and January 2017 were recruited for the study. Patients were included if they had received trastuzumab as their neoadjuvant treatment; had complete data including clinicopathologic features, therapy management, and therapy response; if the planned therapy was completed; and if the pathology specimens obtained were deemed sufficient for YAP, AKT, p-AKT, and p73 analysis of their pre- and post-treatment tissue pathology specimens. Patients were treated according to two regimens - 23 patients were treated with paclitaxel, doxorubicin, and cyclophosphamide (TAC) neoadjuvant chemotherapy, and 14 were treated with docetaxel, carboplatin, and trastuzumab (TCbH) neoadjuvant chemotherapy.

Pathological complete response (pCR) was defined as those samples with no residual invasive lesion in both breast and axilla (ypT0/isN0), as assessed by two pathologists. The local tumor response to the treatment protocol was evaluated using the response evaluation criteria in solid tumors (RECIST) and MRI volumetric assessment. According to the RECIST criteria, complete response (CR) was defined as the complete disappearance of all recognizable tumors in the breast confirmed 4 weeks after the procedure. Partial response (PR) was defined as a reduction of at least 30% in the sum of the longest diameter of the lesions, taking as reference the baseline study, and was confirmed after four weeks. Stable disease (SD) was defined when neither the PR criteria nor the progressive disease criteria were met, taking as reference the smallest sum of the longest diameter recorded since the start of the treatment. Progressive disease (PD) was defined as the appearance of new lesions or as a minimum 20% increase in the sum of the longest diameter of the lesions, taking as reference the smallest sum of the longest diameter recorded from the initiation of treatment 33.

### Statistical analyses

All statistical analyses were performed using SPSS statistical software, version 21.0 (SPSS Inc, Chicago, IL, USA). Continuous variables were evaluated using the Student's *t*-test or Mann-Whitney U-test, as appropriate, and categorical variables were analyzed using the chi-square test. Data from biological triplicate experiments are presented with error bars representing the mean ± standard deviation (SD) unless otherwise indicated, and *P* < 0.05 was deemed statistically significant.

## Results

### YAP expression in breast carcinoma cells

**Figure 1:**
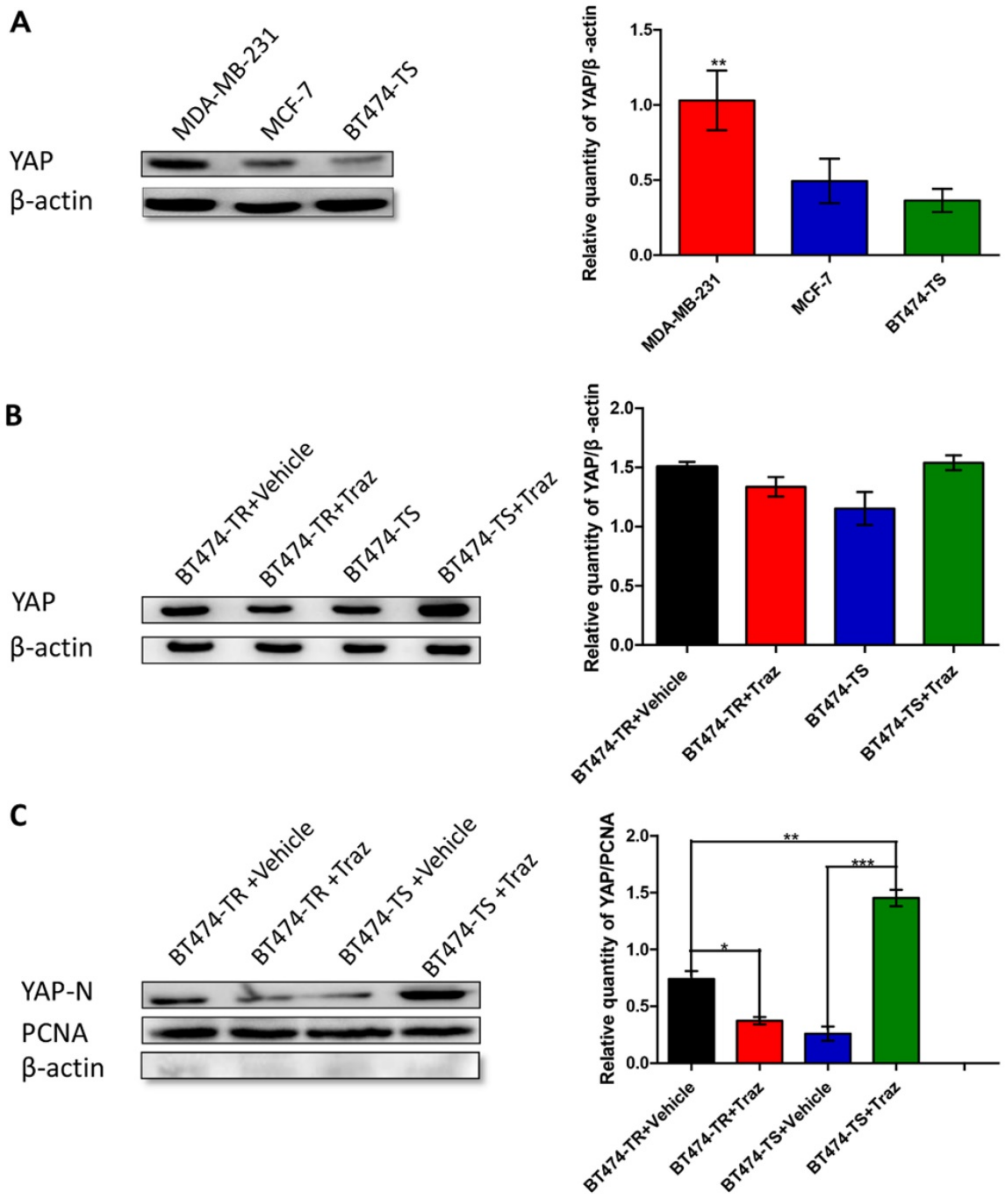
YAP expression in various cell lines. Trastuzumab (Traz) treatment affected YAP expression in BT474-TS and BT474-TR cells. (A) YAP protein expression was detected by western blot in different cancer cells. (B) Total YAP expression after trastuzumab treatment in BT474-TS and BT474-TR cells. (C) Trastuzumab treatment significantly affected N-YAP levels in BT474-TS and BT474-TR cells. Data represent the mean ± standard deviation (SD) of three independent experiments. *p < 0.05, **p < 0.01, and *** p < 0.001.

We have previously demonstrated that YAP expression was inversely associated with HER2 status in breast cancer tissues 14. Therefore, to further study YAP expression in breast carcinoma cells, particularly in the breast carcinoma cell lines MDA-MB-231, MCF-7, and BT474-TS, we performed western blot analysis. YAP was found to be differently expressed in the three cell lines, with the highest level observed in MDA-MB-231 cells, a triple-negative breast cancer cell line, and the lowest in BT474-TS cells (Fig. 1A).

To determine the optimal treatment dosage, we examined the response of BT474-TS cells to treatment with different concentrations of trastuzumab for 96 h using the CCK-8 assay (Fig. S1). As in previous studies, a concentration of 10 μg/mL was chosen for use in subsequent experiments. We then analyzed the effects of treatment time on BT474-TS cell viability (Fig. S2). The highest inhibition ratio was detected following a 48-h treatment, which was, therefore, chosen as the treatment duration in subsequent experiments.

We then examined the effects of trastuzumab administration on YAP expression levels in BT474-TS and BT474-TR cells. Results demonstrated that both BT474-TS and BT474-TR cells expressed comparable levels of YAP (Fig. 1B). However, trastuzumab markedly increased N-YAP levels in BT474-TS cells, while inhibiting N-YAP levels in BT474-TR cells (Fig. 1C).

### YAP-knockdown decreases trastuzumab-induced apoptosis in HER2-positive breast carcinoma cells

**Figure 2:**
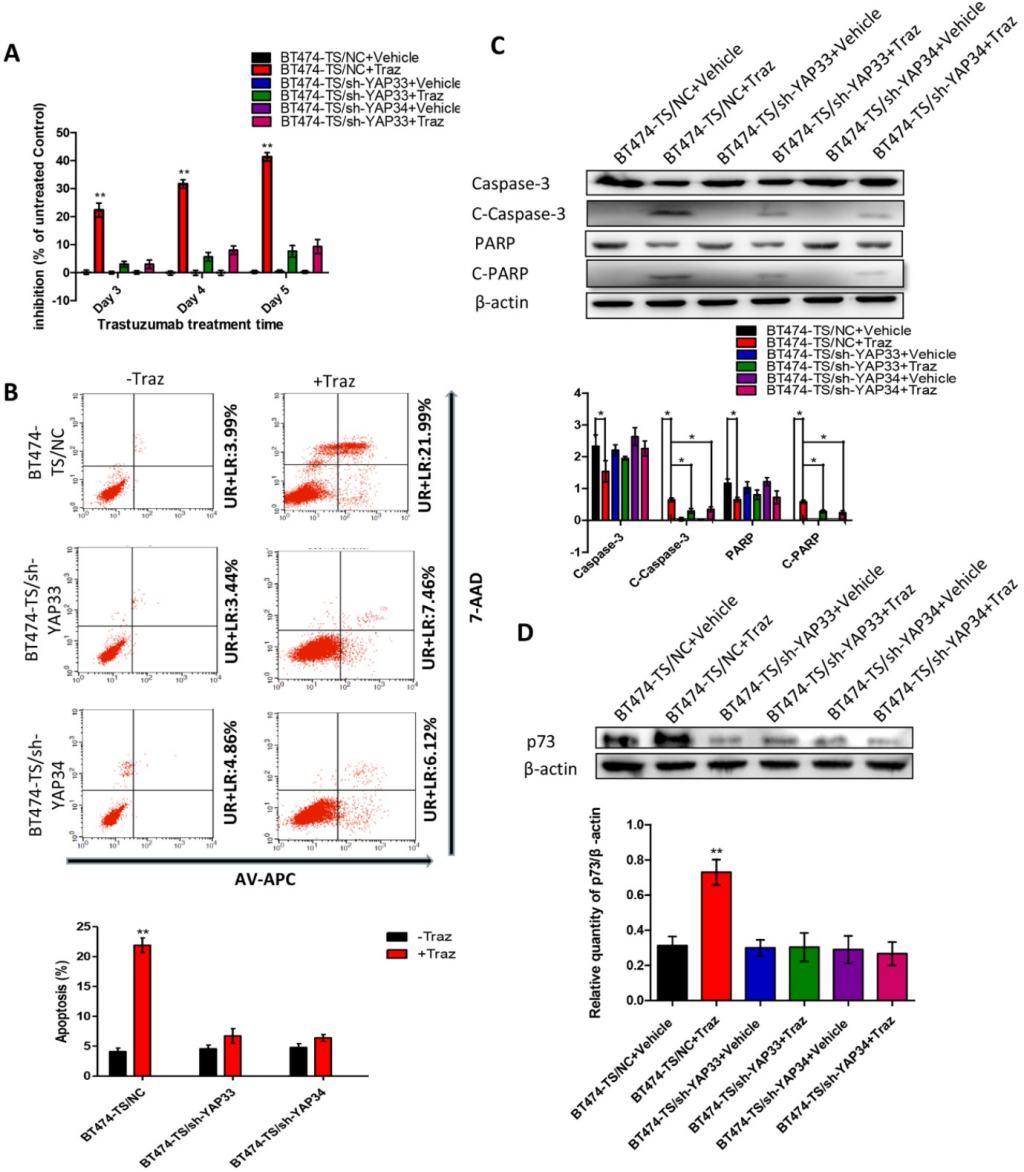
YAP-knockdown altered trastuzumab (Traz) treatment effects and p73 expression in BT474-TS cells. (A) YAP-knockdown increased viability of BT474-TS cells after trastuzumab treatment. (B) YAP-knockdown altered apoptosis, measured by flow cytometry, after treatment in BT474-TS cells. (C) Expression of apoptotic proteins was examined by western blotting. (D) p73 protein expression was detected by western blotting in different cancer cells. Data represent the mean ± standard deviation (SD) of three independent experiments. *p < 0.05, **p < 0.01, and *** p < 0.001.

To test the potential roles of YAP in trastuzumab resistance, we designed and tested four distinct shRNAs against YAP and identified two that effectively knocked down YAP in BT474-TS cells (Fig. S3). To further determine whether the depletion of YAP would confer trastuzumab resistance to BT474-TS cells, we evaluated the inhibition of BT474-TS cells infected with either shGFP or shYAP in mock vs. trastuzumab treatments. BT474-TS cells infected with shGFP demonstrated a marked response to trastuzumab; however, the ratio of inhibition in shYAP-infected BT474-TS cells treated with trastuzumab decreased (Fig. 2A). We also examined apoptosis in BT474-TS cells in response to shRNA-mediated depletion of YAP and detected a significant decrease in apoptosis in BT474-TS cells treated with trastuzumab and infected with shYAP compared to that infected with shGFP (Fig. 2B). Additionally, in the YAP-silenced group, tumor cell apoptosis was decreased following treatment with trastuzumab, and the levels of cleaved caspase-3 and PARP expression were significantly decreased (Fig. 2C). Together, these findings demonstrate that YAP depletion partially conferred trastuzumab resistance to BT474-TS cells.

### YAP promotes p73-induced apoptosis

AKT attenuates p73-mediated apoptosis by phosphorylating YAP and inducing its interaction with 14-3-3 in Cos-7 cells 34. Therefore, to further verify the hypothesis that the AKT/YAP/p73 pathway compensates for HER2 inhibition by trastuzumab, we subsequently performed the following experiments (Fig. S4).

**Figure 3:**
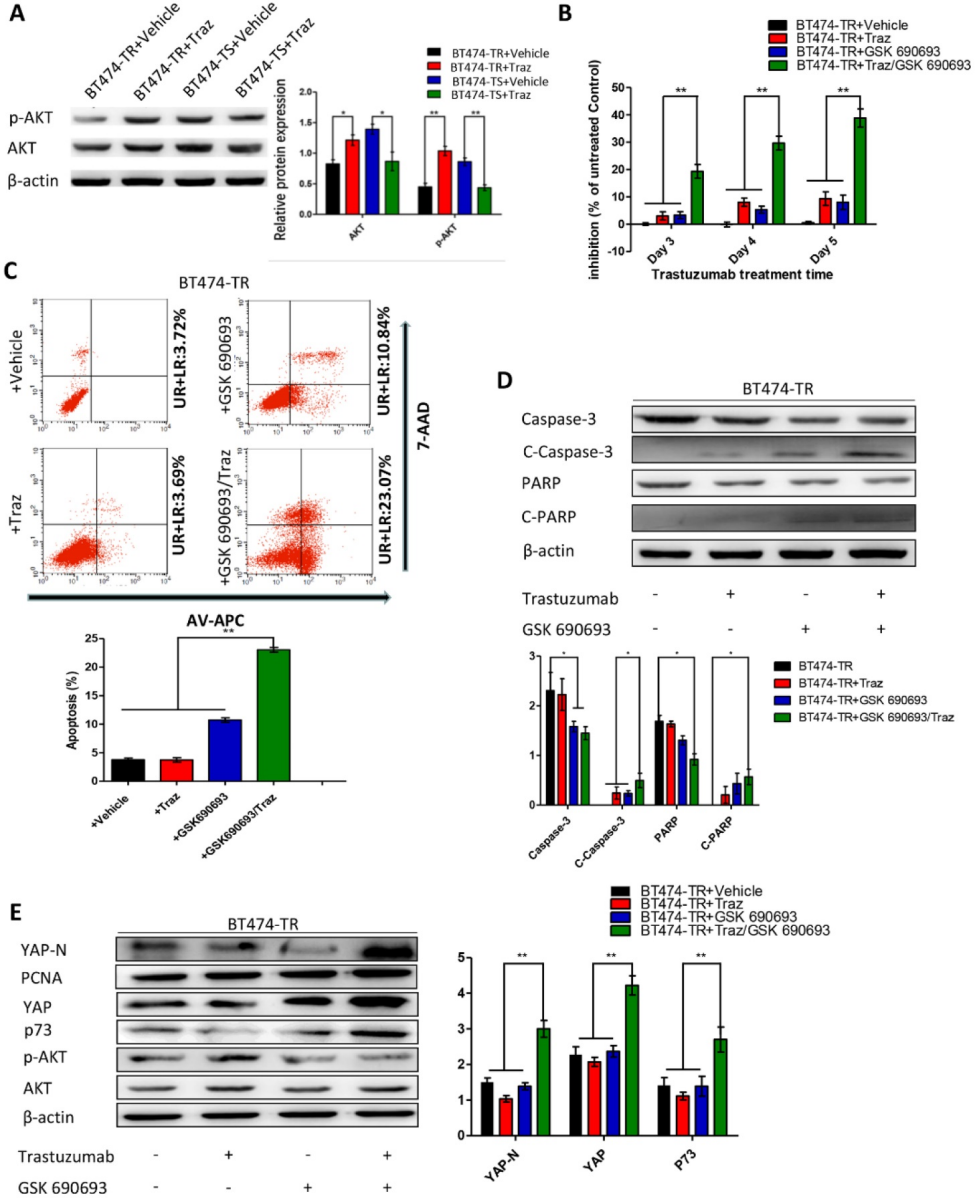
Trastuzumab (Traz) treatment affected AKT and p-AKT expression levels in BT474-TS and BT474-TR cells. Combination treatment of AKT inhibitor (GSK) and Traz in BT474-TR cells. (A) Trastuzumab treatment significantly affected AKT and p-AKT levels in BT474-TS and BT474-TR cells. (B) Trastuzumab plus GSK strongly affected the viability of BT474-TR cells. (C) Trastuzumab plus GSK increased apoptosis ratio of BT474-TR cells; (D) Expression of apoptotic proteins were examined by western blotting; (E) YAP, N-YAP, and p73 expression in BT474-TR cells after combination treatment. Data represent the mean ± standard (SD) of three independent experiments. *p < 0.05 and **p < 0.01.

First, we evaluated p73 levels in BT474-TS cells infected with either shGFP or shYAP in mock vs. trastuzumab treatments. Trastuzumab did not induce p73 expression in BT474-TS cells expressing shYAP (Fig. 2D). We then examined AKT and p-AKT levels in BT474-TS and BT474-TR cells and found that AKT and p-AKT expression levels were markedly increased in BT474-TR cells and decreased in BT474-TS cells following trastuzumab treatment (Fig. 3A).

We further examined the effects of GSK 690693 on YAP and p73 in BT474-TR cells as follows: BT474-TR cells were treated with trastuzumab either alone or in combination with GSK 690693, and the levels of YAP, N-YAP, and p73 were measured by western blot. Results demonstrate that trastuzumab with the addition of GSK 690693 was sufficient to re-induce N-YAP and p73 protein levels (Fig. 3E).

Furthermore, we overexpressed YAP in the BT474-TR cell line and detected whether its overexpression could attenuate trastuzumab resistance. Results show that apoptosis and the levels of cleaved caspase-3 and PARP expression were significantly increased by combined treatment with trastuzumab and GSK 690693 of YAP-overexpressing BT474-TR cells (Fig. S5). Additionally, we examined whether the overexpression of p73 in YAP-silenced BT474-TS cells could rescue the effects mediated by YAP-knockdown. Results demonstrate that apoptosis, as well as the levels of cleaved caspase-3 and PARP expression, were significantly increased by combined treatment with trastuzumab and p73 overexpression of YAP-silenced BT474-TS cells (Fig. S6).

These findings demonstrate that the aberrant activation of AKT in breast carcinoma cells inhibits apoptosis induced by YAP-p73 and attenuates the inhibitory effects of trastuzumab on tumor cells, resulting in development of trastuzumab resistance.

### p-AKT and YAP as biomarkers of tumor response in tissues of patients with breast cancer receiving neoadjuvant therapy

The 37 patients with invasive breast cancer included in this study had a median age of 55 years (range: 29-86 years); three (13.0%) patients achieved a pCR in the TAC group, and four (28.6%) achieved a pCR in the TCbH group.

Local tumor evaluation according to RECIST criteria were as follows: among the 23 HER2 negative TAC-treated patients, 5 (21.7%) achieved CR, 7 (30.4%) achieved PR, 9 (39.1%) had SD, and 2 (10.6%) had PD. Among the 14 HER2 positive TCbH-treated patients, 4 (28.6%) achieved CR, none achieved PR, 1 (7.1%) had SD, and 9 (62.3%) had RD.

**Figure 4:**
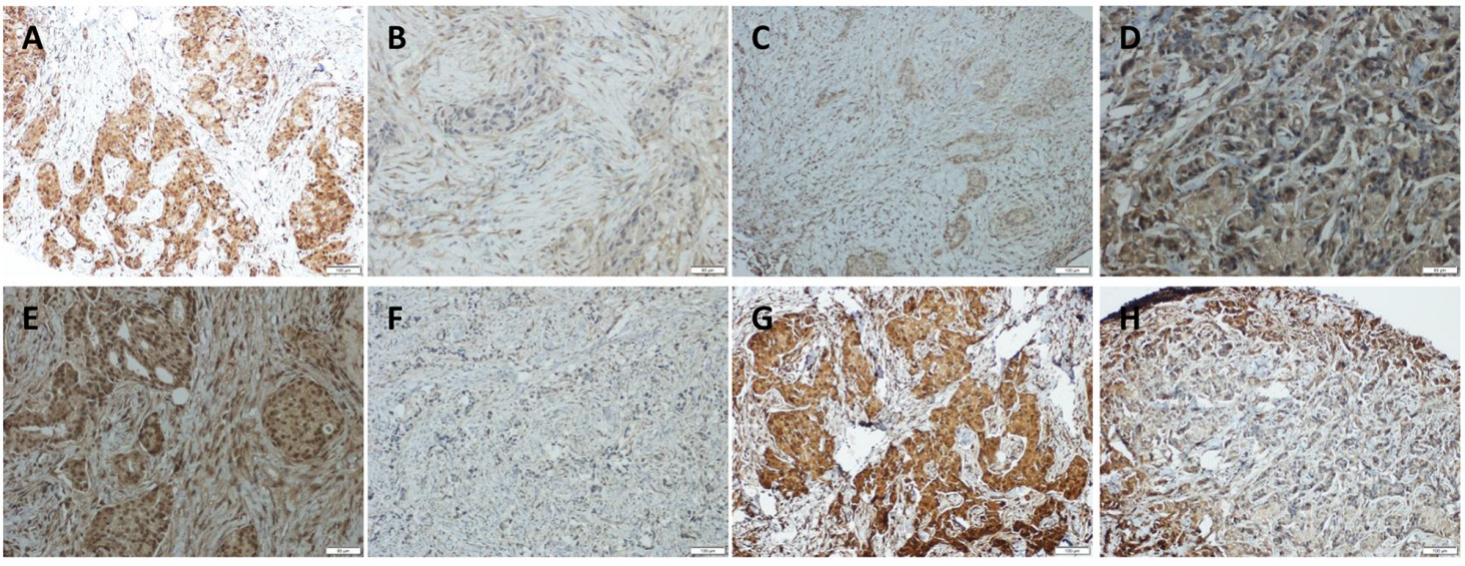
Immunohistochemical detection of YAP, p73, AKT, and p-AKT expression in pre-treated HER2-positive breast cancer tissues. (A and B) Positive and negative expression of YAP in breast cancer tissues. (C and D) Positive and negative expression of p73 in breast cancer tissues. (E and F) Positive and negative expression of AKT in breast cancer tissues. (G and H) Positive and negative expression of p-AKT in breast cancer tissues (original magnification ×100).

To further investigate the correlation among YAP, AKT, and p73, we immunolocalized YAP, AKT, p-AKT, and p73 in 23 TAC, and 14 TCbH pre-treatment biopsies and post-treatment surgical pathology specimens. YAP, AKT, p-AKT, and p73 were observed to be primarily localized in the tumor cell nuclei (Fig. 4A-H). Moreover, p-AKT immunoreactivity was significantly lower in post-treatment surgical specimens than in corresponding biopsy specimens (*p* = 0.010, Table 1). YAP expression positively correlated with p73 (*p* = 0.049, *p* = 0.001); however, inversely correlated with p-AKT (*p* = 0.016, *p* < 0.001) in biopsy specimens of both the TAC and TCbH groups (Table S1). In addition, YAP was positively associated with p73 in surgical specimens of the TCbH group (*p* = 0.002), whereas YAP inversely associated with p-AKT in surgical specimens of both the TAC and TCbH groups (*p* = 0.001 and *p* = 0.002, respectively; Table S2), which excluded pCR cases.

In both TAC and TCbH groups, pCR ratios were inversely correlated with p-AKT in biopsy specimens (*p* = 0.005, *p* = 0.018) (Table 2). In addition, YAP and p73 immunoreactivity in biopsy specimens was positively associated with pCR ratio in the TCbH group (*p* = 0.018, *p* = 0.040, respectively; Table 2). Additionally, the YAP expression level was significantly higher in the pCR group than in the non-pCR group, according to western blot analysis (Fig. S7). YAP and p-AKT expression in TCbH groups correlated with local tumor response (*p* = 0.050, *p* = 0.050, respectively; Table 2).

## Discussion

Trastuzumab is widely used in patients with HER2-positive breast cancer and as neoadjuvant therapy for patients with early-stage HER2-positive breast cancer 35, 36. However, the development of therapeutic resistance and low objective response ratios have been reported 2, prompting us to identify novel approaches to improve the therapeutic efficacy of trastuzumab treatment in both trastuzumab-resistant and unresponsive patients. Herein, we demonstrated the following findings: (1) trastuzumab inhibits BT474-TS cell survival by reducing the levels of AKT phosphorylation and also by enhancing YAP-p73-induced apoptosis; (2) the increased AKT phosphorylation in BT474-TR cells diminishes the efficacy of trastuzumab; and (3) both AKT and YAP may serve as effective predictors of pCR ratios in patients undergoing neoadjuvant chemotherapy with trastuzumab.

YAP plays pivotal roles in various human malignancies 37. Generally, it has been shown to function as an oncogene in many cancers; however, studies have also indicated that YAP functions as a tumor suppressor in certain human malignancies, including head and neck 38, colorectal 39, hematological 40, and breast cancers 23, 24. However, the link between YAP and trastuzumab resistance in HER2-positive breast carcinoma cells is not well demonstrated. Therefore, we first detected the expression of YAP and found that YAP-knockdown decreased the therapeutic efficacy of trastuzumab in HER2-positive breast carcinoma cells. Nevertheless, previous studies reported that YAP depletion increases sensitivity to anti-HER2 treatment in breast cancer 26, 41. Indeed, YAP has been implicated as an oncogene in conjunction with the transcriptional enhancer activator domain (TEAD) family of transcription factors in human cancers, where it activates pro-proliferative and antiapoptotic target genes. However, a twist was introduced into this coherent picture when YAP was recognized as a coactivator of proapoptotic genes. In response to DNA damage, YAP targets p73, a member of the tumor suppressor p53 family, to induce proapoptotic gene expression and initiate a cellular death axis, corroborating previous findings 42. Moreover, c-Abl influences YAP behavior in myriad of ways, from inducing the YAP-TEAD survival axis to promoting the opposing function.

Numerous crosstalk events have been described between the ER and HER2 pathways that contribute to the development of therapy resistance to breast cancer treatment 43. Moreover, trastuzumab efficacy is reduced in patients with high levels of ER expression following upregulation of ER pathways, which questions trastuzumab treatment efficacy in “triple positive” breast cancer 44, 45. Consequently, cross talk between ER and HER2 pathways often upregulates one pathway, while the other is inhibited 46. Recently, YAP/TEAD, which are ERa cofactors, were shown to regulate enhancer activation, gene transcription, and breast cancer growth 47. However, when c-Abl becomes activated in response to DNA damage, the tyrosine-phosphorylated YAP continues to bind TEAD without inducing the survival axis 42. This process fully abrogates YAP oncogenic activity. Thus, similar to how modified YAP induces the death axis via p73, activated c-Abl converts YAP from an oncogene to a tumor suppressor. Results of the current study demonstrate that trastuzumab treatment reduced p73 expression in YAP-knockdown BT474-TS cells as compared to that in controls, indicating that YAP-knockdown could attenuate the effects of trastuzumab on the p73-induced apoptosis pathway in HER2-positive breast carcinoma cells. Furthermore, caspase-3 and PARP cleavage, as well as apoptosis ratios, were reduced in the YAP-knockdown groups compared with those in the control groups, indicating that YAP plays a pivotal role in trastuzumab-induced tumor cell apoptosis.

The mechanisms underlying the effects of trastuzumab resistance on HER2-positive carcinoma cells have been reported to be primarily associated with the intracellular AKT pathway 9, 36, 48. AKT promotes YAP loss from the nucleus, where it functions as a coactivator of transcription factors and mediates the activation of pro-apoptosis genes, including p73 34, which is also consistent with the results of our present study. We showed that trastuzumab treatment increased AKT phosphorylation (Ser473) in BT474-TR cells and that combined treatment with trastuzumab and GSK increased N-YAP expression in BT474-TR cells as compared to that in the controls. Further, these findings indicate that YAP-knockdown attenuated the effects of trastuzumab on AKT in HER2-positive breast carcinoma cells. The AKT pathway is an important proliferative pathway, while AKT reactivation serves as an important mechanism for resistance to trastuzumab treatment 8, 9. Our results show that trastuzumab treatment increased AKT phosphorylation levels in BT474-TR cells, which subsequently inhibited YAP nucleation and YAP-p73 mediated apoptosis. Moreover, the levels of N-YAP were higher in the resistant cell line. Thus, AKT may influence not only the localization of YAP, but also the target gene of YAP in stable conditions. Alternatively, other proteins upstream of YAP such as c-Abl 42 may have also altered YAP function. Hence, additional *in vitro* and *in vivo* studies are warranted to confirm these speculations.

Our results also demonstrate that treatment with trastuzumab and GSK 690693, an AKT inhibitor, significantly inhibited AKT phosphorylation, while promoting YAP nucleation, and significantly induced apoptosis in BT474-TR cells. AKT activation was previously shown to promote YAP-14-3-3 binding, which not only reduces YAP nucleation, but also leads to YAP degradation, thereby inhibiting YAP-p73 apoptosis 34, which is in line with our results. Hence, trastuzumab and GSK 690693 treatment combination not only increased the levels of YAP nucleation, but also affected YAP expression. Moreover, as studies have shown that overexpression of CD147 49, S100-P 50, or TTK 51 can promote AKT activation and lead to trastuzumab resistance, these oncogenes may also act as upstream molecules of AKT-YAP to protect against trastuzumab inhibition.

We previously reported that YAP is a prognostic factor in breast cancer patients 14. Our present results confirm that YAP may serve as a clinically significant predictor of disease prognosis and response to trastuzumab-based neoadjuvant chemotherapy in patients with breast cancer. Further, we revealed the following novel aspects. First, we evaluated and compared AKT, p-AKT, YAP, and p73 status between pre-treatment biopsies and post-treatment surgical specimens. In the TAC group, p-AKT status was decreased significantly after treatment, which was supported by the results of a previous study demonstrating that the PTEN/PI3K/AKT/mTOR pathway is involved in the development of trastuzumab resistance 48. Results from our present study demonstrate that YAP and p-AKT expression serve as predictors of the effects of neoadjuvant chemotherapy and suggest the possibility of combining AKT inhibitor and trastuzumab treatment for high-resistance-risk breast cancer; however, further investigations are required for clarifying this promising hypothesis.

Our study has several limitations. First, this was a single-institution retrospective study in which small numbers of patients who underwent neoadjuvant chemotherapy were enrolled and, therefore, potential selection biases cannot be ruled out. Second, more detailed underlying mechanisms of YAP in trastuzumab resistance are yet to be elucidated; however, our current study could potentially offer novel mechanistic insights and therapeutic targets to the field of cancer biology. Finally, our study primarily focused on *in vitro* studies; therefore, additional *in vitro* studies with HER2-positive cell lines as well as additional *in vivo* studies are warranted to confirm the findings of the current study.

In summary, our study revealed a potential mechanism underlying trastuzumab resistance and demonstrated the predictive utility of YAP and p-AKT in trastuzumab-based neoadjuvant chemotherapy responses in patients with breast cancer. Our results may provide insights for improving the efficacy of trastuzumab treatment in patients with HER2-positive breast cancer. Accordingly, additional studies are needed to confirm the clinical efficacy of AKT inhibitors as single agents or in combination with trastuzumab and neoadjuvant chemotherapy.

## Supplementary Material

Supplementary figures and tables.

## Figures and Tables

**Table 1:** YAP, p73, AKT, and p-AKT expression in cancer tissues of non-pCR patients before and after neoadjuvant therapy

TAC		No.	Neoadjuvant therapy		*p*-value	TCbH		No.	Neoadjuvant therapy		*p*-value
			Pre-treatment	Post-treatment					Pre-treatment	Post-treatment	
			No. (%)	No. (%)					No. (%)	No. (%)	
YAP	negative	23	12 (60.0)	11 (55.0)	0.749	YAP	negative	11	7 (63.6)	4 (36.4)	0.178
	positive	17	8 (40.0)	9 (45.0)			positive	9	3 (33.3)	6 (66.7)	
p73	negative	14	8 (40.0)	6 (30.0)	0.507	p73	negative	10	6 (60.0)	4 (40.0)	0.371
	positive	26	12 (60.0)	14 (70.0)			positive	10	4 (40.0)	6 (60.0)	
AKT	negative	10	3 (15.0)	7 (35.0)	0.144	AKT	negative	6	3 (50.0)	3 (50.0)	1
	positive	30	17 (85.0)	13 (65.0)			positive	14	7 (50.0)	7 (50.0)	
p-AKT	negative	16	4 (20.0)	12 (60.0)	0.01	p-AKT	negative	9	3 (33.3)	6 (66.7)	0.178
	positive	24	16 (80.0)	8 (40.0)			positive	11	7 (63.6)	4 (36.4)	

Abbreviations: YAP, Yes-associated protein; TAC, paclitaxel, doxorubicin, and cyclophosphamide; TCbH, docetaxel, carboplatin, and trastuzumab.

**Table 2:** Association between treatment response and expression of YAP, p73, AKT, and p-AKT in cancer tissues of patients who received neoadjuvant therapy

			No.	pCR		*p*-value	Response (RECIST)				*p*-value
				Non-pCR	pCR		CR	PR	SD	PD	
				No. (%)	No. (%)		No. (%)	No. (%)	No. (%)	No. (%)	
TAC	YAP	negative	12	12 (100)	0 (0)	0.052	1 (8.3)	5 (41.6)	5 (41.6)	1 (8.3)	0.218
		positive	11	8 (72.7)	3 (27.3)		4 (36.4)	2 (18.2)	4 (36.4)	1 (9)	
	p73	negative	9	8 (88.9)	1 (11.1)	0.825	2 (22.2)	2 (22.2)	5 (55.6)	0 (0)	0.600
		positive	14	12 (85.7)	2 (14.3)		3 (21.4)	4 (28.6)	5 (35.7)	2 (14.3)	
	AKT	negative	4	3 (75.0)	1 (25.0)	0.435	1 (25)	0 (0)	2 (50)	1 (25)	0.421
		positive	19	17 (89.5)	2 (10.5)		4 (21.1)	6 (31.6)	8 (42.1)	1 (5.2)	
	p-AKT	negative	7	4 (57.1)	3 (42.9)	0.005	3 (42.8)	1 (14.3)	2 (28.6)	1 (14.3)	0.324
		positive	16	16 (100)	0 (0)		2 (12.5)	5 (31.3)	8 (50)	1 (6.2)	
TCbH	YAP	negative	7	7 (100)	0 (0)	0.018	0 (0)	0 (0)	1 (14.3)	6 (85.7)	0.050
		positive	7	3 (42.9)	4 (57.1)		4 (57.1)	0 (0)	0 (0)	3 (42.9)	
	p73	negative	6	6 (100)	0 (0)	0.04	0 (0)	0 (0)	0 (0)	6 (100)	0.054
		positive	8	4 (50)	4 (50)		4 (50)	0 (0)	1 (12.5)	3 (37.5)	
	AKT	negative	3	3 (100)	0 (0)	0.217	0 (0)	0 (0)	0 (0)	3 (100)	0.346
		positive	11	7 (63.6)	4 (36.4)		4 (36.4)	0 (0)	1 (9.1)	6 (54.5)	
	p-AKT	negative	7	3 (42.9)	4 (57.1)	0.018	4 (57.1)	0 (0)	0 (0)	3 (42.9)	0.050
		positive	7	7 (100)	0 (0)		0 (0)	0 (0)	1 (14.3)	6 (85.7)	

Abbreviations: YAP, Yes-associated protein; pCR, pathological complete response; TAC, paclitaxel, doxorubicin, and cyclophosphamide; TCbH, docetaxel, carboplatin, and trastuzumab.

## Abbreviations

ATCC: American type culture collection
CR: complete response
FBS: fetal bovine serum
FS: final numerical score
GFP: green fluorescent protein
pCR: pathological complete response
PD: progressive disease
PR: partial response
RECIST: response evaluation criteria in solid tumors
SD: stable disease
TEAD: transcriptional enhancer activator domain
YAP: Yes-associated protein
