# Supplementary material for: YAP increases response to Trastuzumab in HER2-positive Breast Cancer by enhancing P73-induced apoptosis
Source: J Cancer. 2020 Sep 25;11(22):6748–59. doi: 10.7150/jca.48535 (PMC7545685; doi:10.7150/jca.48535)
Supplement: Supplementary file 1 — Supplementary figures and tables. [file jcav11p6748s1.pdf]

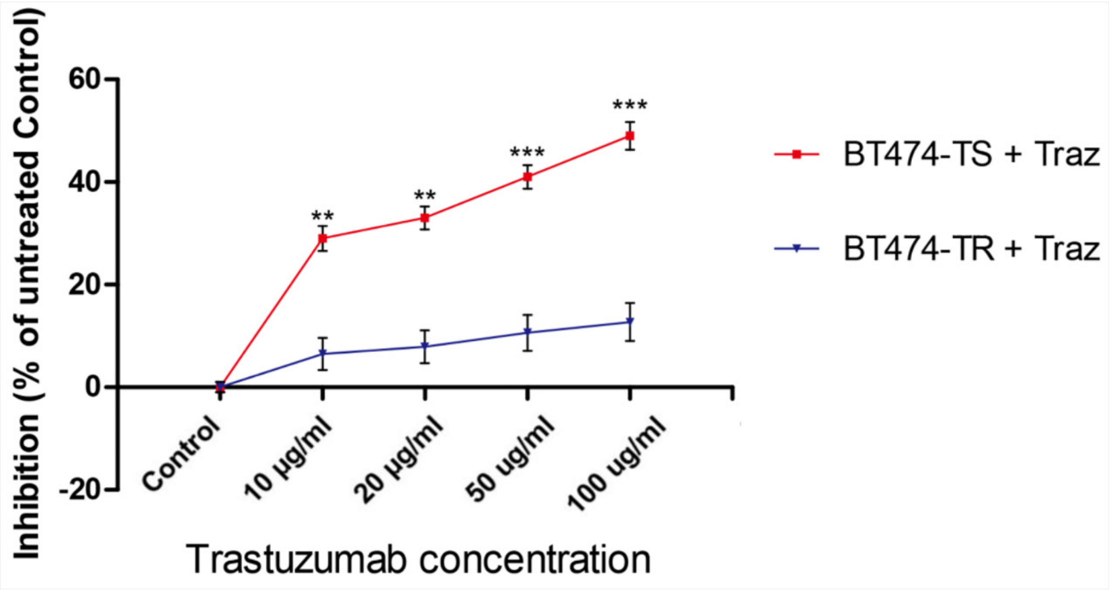

Fig. S1. Inhibition of cell viability by trastuzumab, as measured by cell counting kit-8 assay, occurred in a dose-dependent manner 4 days following treatment with trastuzumab at five different concentrations. Data are presented as mean ± standard deviation (SD). The data shown are representative of three independent experiments. ***$p < 0.001$, **$p < 0.01$.

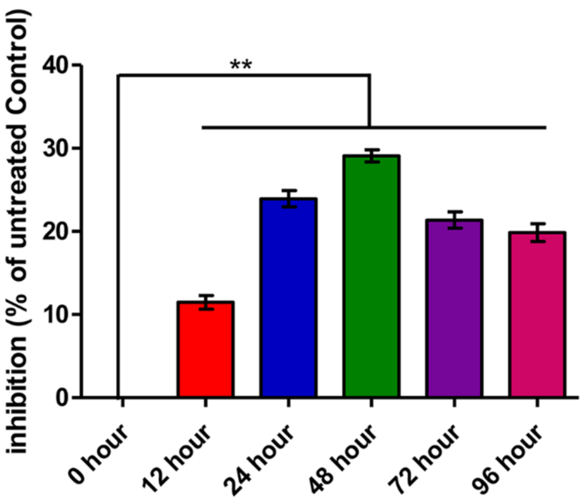

Fig. S2. Trastuzumab (10 μg/mL) inhibited cell viability in a time-dependent manner. Data are presented as mean ± standard deviation (SD). The data shown are representative of three independent experiments. **$p < 0.01$.

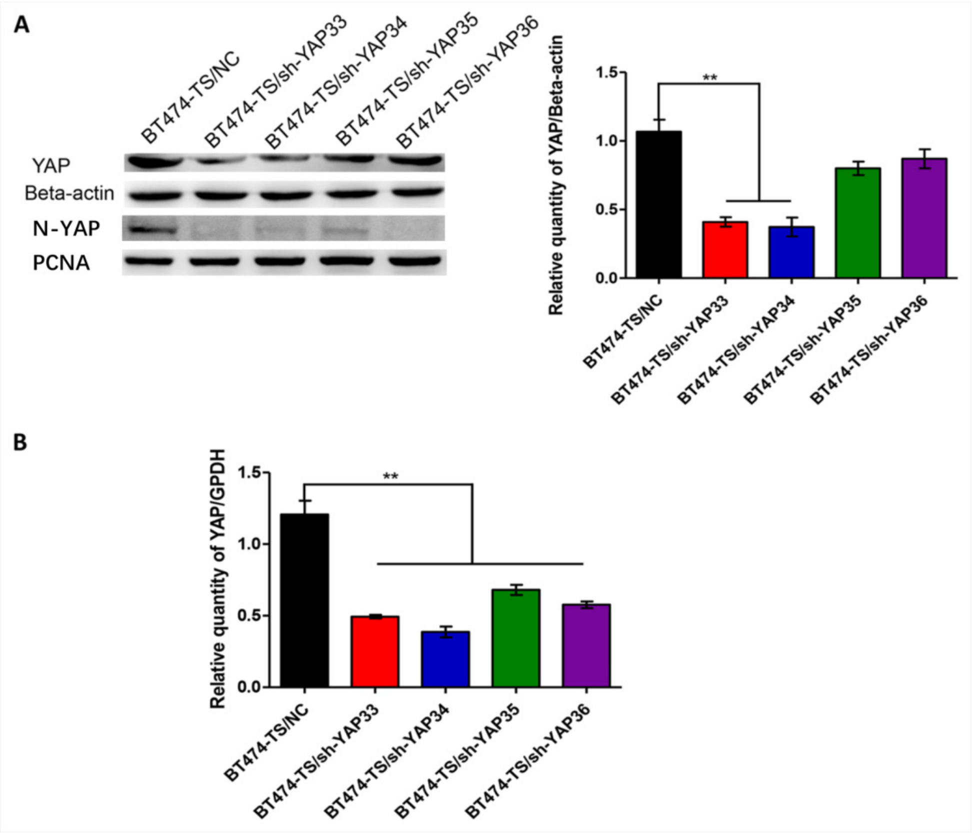

Fig. S3. YAP knockdown. (A) YAP protein and N-YAP expression were detected, using western blotting, in BT474-TS cells. (B) YAP mRNA levels detected, by RT-PCR, in BT474-TS cells. Data are presented as mean ± standard deviation (SD). The data shown are representative results of three independent experiments. $^{**}p < 0.01$.

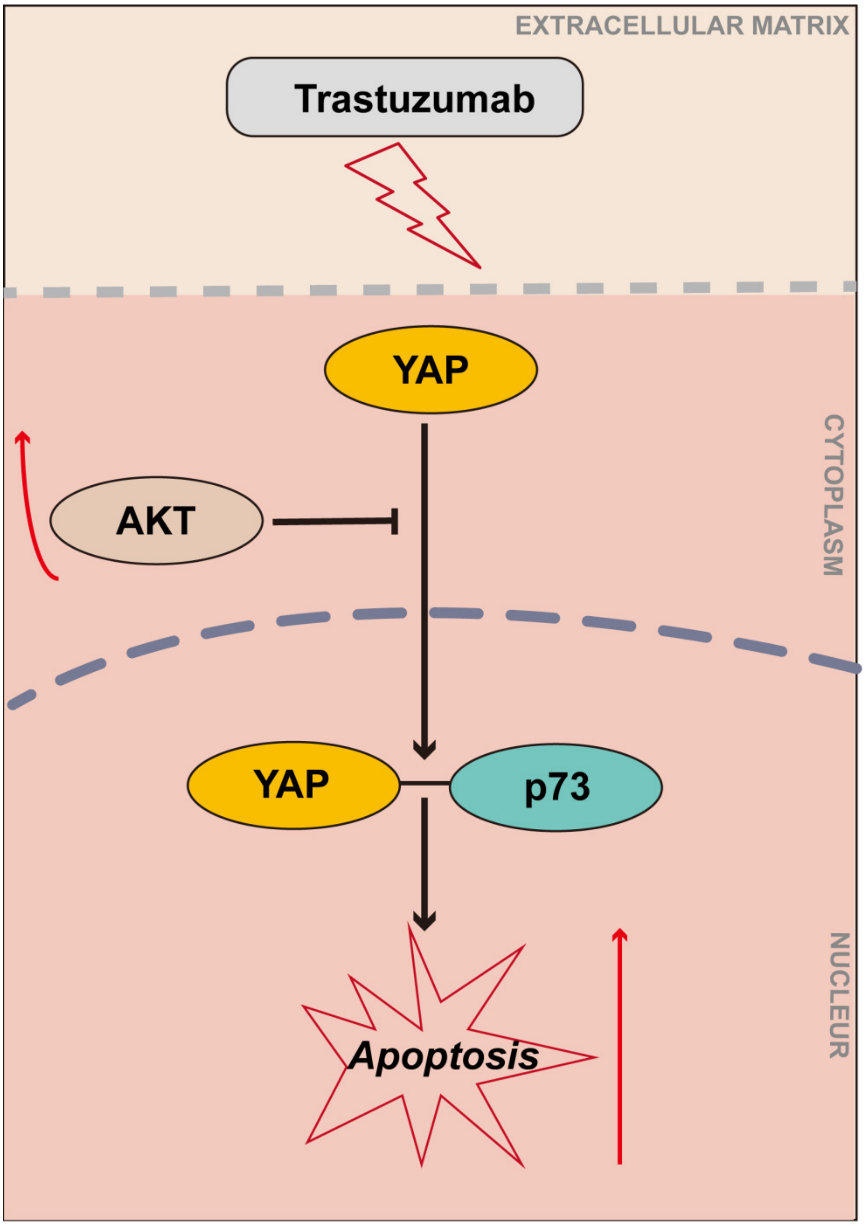

Fig. S4. Study hypothesis: AKT attenuates p73-mediated apoptosis by phosphorylating YAP.

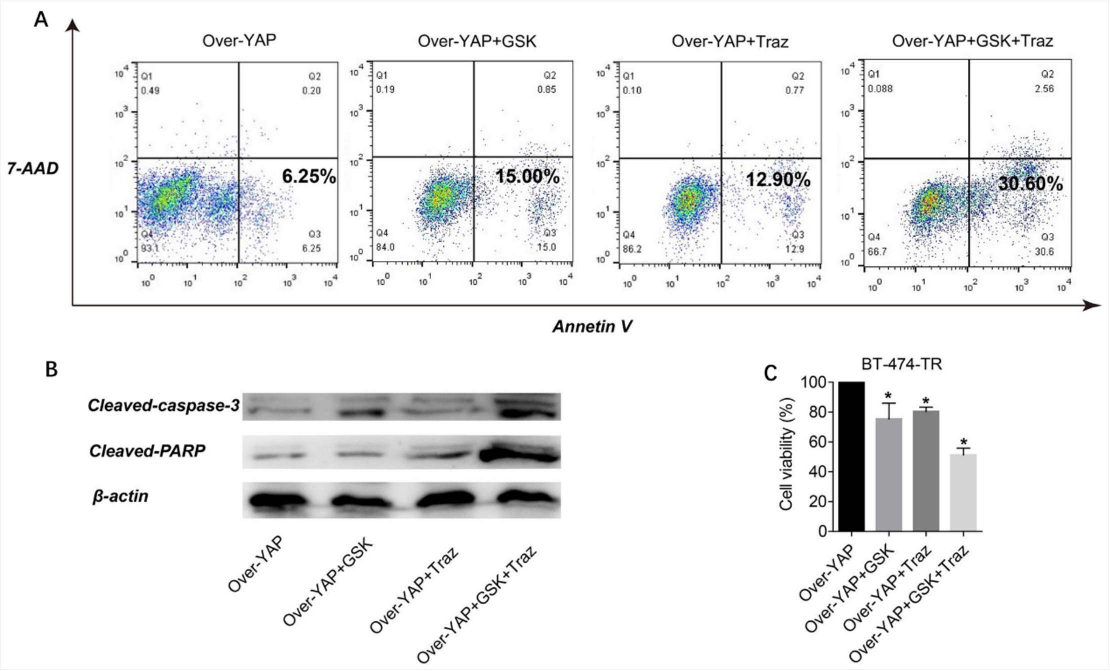

Fig. S5. Combination treatment of AKT inhibitor (GSK) and trastuzumab of YAP-over BT474-TR cells. (A) Trastuzumab plus GSK increased apoptosis rate of BT474-TR cells. (B) Expression of apoptotic proteins was examined using western blotting. (C) Trastuzumab plus GSK strongly affected the viability of BT474-TR cells after YAP-overexpression. Data are presented as mean ± standard deviation (SD). The data shown are representative of three independent experiments. $*p < 0.05$.

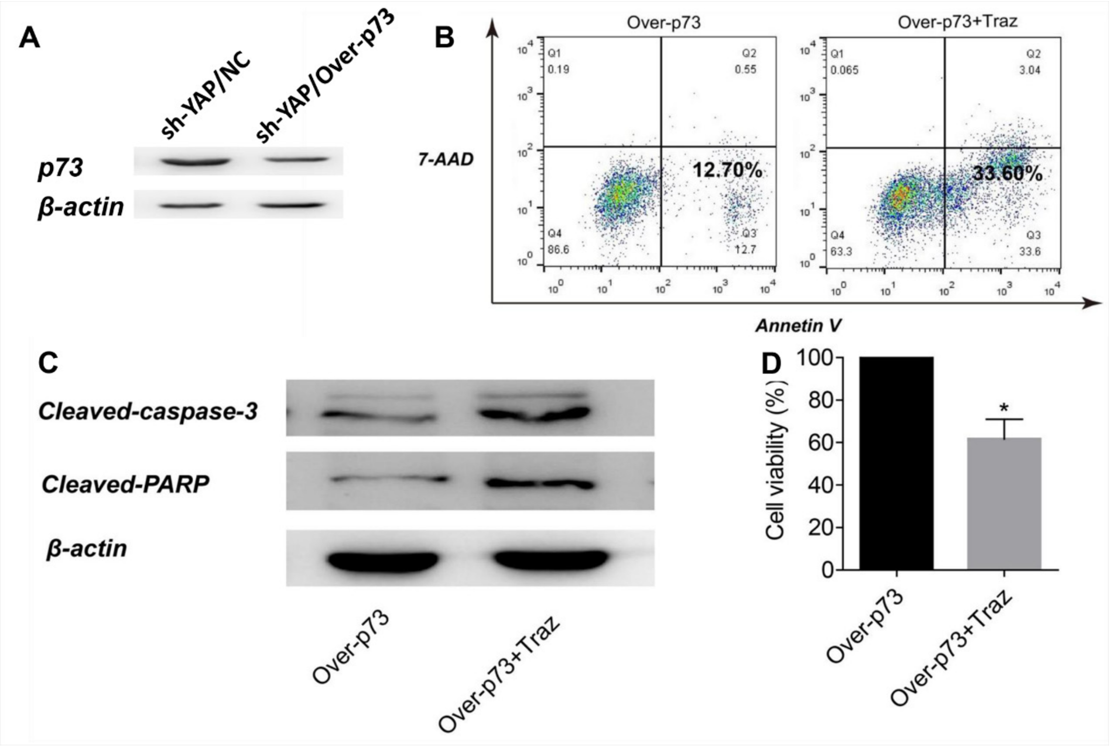

Fig. S6. p73-overexpression altered trastuzumab treatment effects in YAP-silenced BT474-TS cells. (A) p73 protein expression was detected, by western blotting, in YAP-silenced BT474-TS cells. (B) p73-overexpression altered apoptosis, measured by flow cytometry, following treatment of YAP-silenced BT474-TS cells. (C) Expression of apoptotic proteins was examined by western blotting. (D) p73-overexpression decreased the viability of YAP-silenced BT474-TS cells after trastuzumab treatment; Data are presented as mean ± standard deviation (SD). The data shown are representative of three independent experiments. *$p < 0.05$.

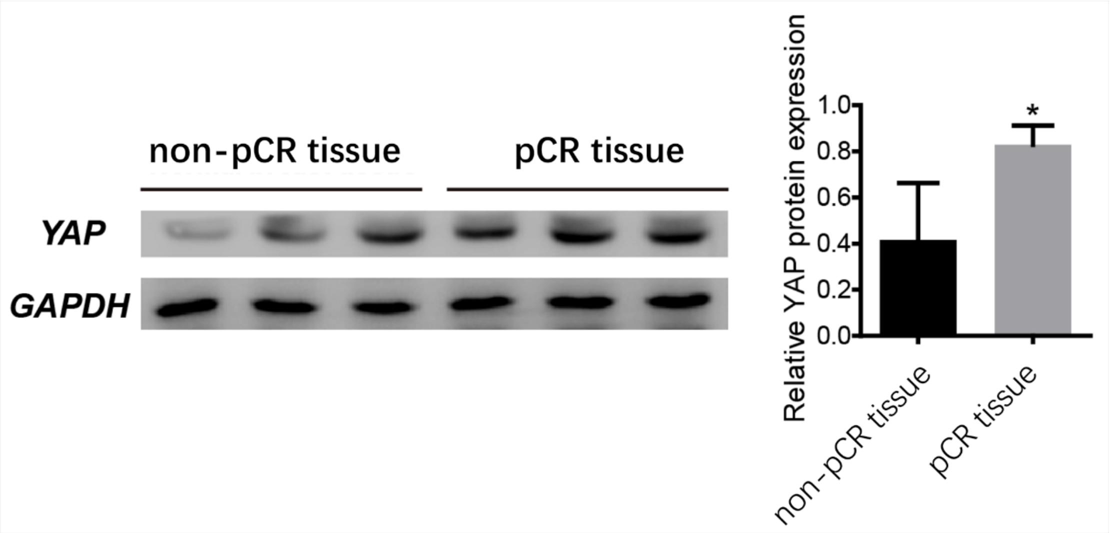

Fig. S7. YAP expression in breast cancer tissues. YAP protein expression was detected, by western blot, in pCR and non-pCR breast cancer tissues. Data are presented as mean ± standard deviation (SD). The data shown are representative of three independent experiments. *p <0.05.

Table S1. Association between YAP and p73, AKT, and p-AKT in pre-treatment biopsy tissues

			No.	YAP		p-value
				Negative *No.* (%)	*Positive* *No.* (%)	
TAC	p73	negative	9	7 (77.8)	2 (22.2)	
		positive	14	5 (35.7)	9 (64.3)	0.049
	AKT	negative	4	1 (25.0)	3 (75.0)	
		positive	19	11 (60.0)	8 (40.0)	0.291
	p-AKT	negative	7	1 (14.3)	6 (85.7)	
		positive	16	11 (68.8)	5 (31.2)	0.016
TCbH	p73	negative	6	6 (100)	0 (0)	
		positive	8	1 (12.5)	7 (87.5)	0.001
	AKT	negative	3	0 (0)	3 (100)	
		positive	11	7 (63.6)	4 (36.4)	0.051
	p-AKT	negative	7	0 (0)	7 (100)	
		positive	7	7 (100)	0 (0)	< 0.001

Table S2. Association between YAP and p73, AKT, and p-AKT in post-treatment surgical tissues

			No.	YAP		*p*-value
				Negative *No.* (%)	*Positive* *No.* (%)	
TAC	p73	negative	6	5 (83.3)	1 (16.7)	
		positive	14	6 (42.9)	8 (57.1)	0.095
	AKT	negative	10	3 (30.0)	7 (70.0)	
		positive	13	8 (61.5)	5 (38.5)	0.133
	p-AKT	negative	12	3 (25.0)	9 (75.0)	
		positive	8	8 (100)	0 (0)	0.001
TCbH	p73	negative	4	4 (100)	0 (0)	
		positive	6	0 (0)	6 (100)	0.002
	AKT	negative	3	0 (0)	3 (100)	
		positive	7	4 (57.1)	3 (42.9)	0.091
	p-AKT	negative	6	0 (0)	6 (100)	
		positive	4	4 (100)	0 (0)	0.002
